# A Rare Case of Pure Erythroid Sarcoma in a Pediatric Patient: Case Report and Literature Review

**DOI:** 10.3390/children4120113

**Published:** 2017-12-20

**Authors:** Pablo Manresa, Fabián Tarín, María Niveiro, María Tasso, Olga Alda, Francisco López, Héctor Sarmiento, José J. Verdú, Francisco De Paz, Silvia López, María Del Cañizo, Esperanza Such, Eva Barragán, Fernanda Martirena

**Affiliations:** 1 Department of Hematology, Hospital General Universitario de Alicante, 03010 Alicante, Spain; tarin_fab@gva.es (F.T.); olgaaldaal@gmail.com (O.A.), flopcas@hotmail.com (F.L.); h.sarmiento2475@gmail.com (H.S.); pepeverd@hotmail.com (J.J.V.); depaz_fra@gva.es (F.D.P.); 2 Department of Anatomic Pathology, Hospital General Universitario de Alicante, 03010 Alicante, Spain; mniveiro@gmail.com; 3 Department of Pediatrics, Hospital General Universitario de Alicante, 03010 Alicante, Spain; tasso_mar@gva.es (M.T.); silvialoncologia@gmail.com (S.L.); mariacmoreira113@gmail.com (M.D.C.); 4 Department of Hematology, Hospital Universitario La Fe de Valencia, 46009 Valencia, Spain; such_esp@gva.es (E.S.); barragan_eva@gva.es (E.B.); 5 Department of Hematology, Hospital de Elda, 03600 Elda, Alicante, Spain; fmartirena@gmail.com

**Keywords:** red blood cell disorders, leukemias, acute, hematology, immunophenotype, sarcomas, including soft tissue, non-rhabdoid

## Abstract

We describe an exceptional case of erythroid sarcoma in a pediatric patient as a growing orbital mass with no evidence of morphologic bone marrow involvement, who was finally diagnosed of pure erythroid sarcoma based on histopathology and flow cytometry criteria. We discuss the contribution of standardized eight-color flow cytometry as a rapid and reliable diagnostic method. The use of normal bone marrow databases allowed us to identify small aberrant populations in bone marrow and later confirm the diagnosis in the neoplastic tissue.

## A Rare Case of Pure Erythroid Sarcoma in a Pediatric Patient: Case Report and Literature Review

Myeloid sarcoma (MS), previously known as granulocytic sarcoma or chloroma, is an uncommon myeloid neoplasm consisting of a malignant proliferation derived from myeloid hematopoietic blasts occurring in an anatomical site other than bone marrow (BM) [1]. MS may precede, follow, or occur in the absence of systemic acute myeloid leukemia in the context of diagnosis or relapse in any body part [2]. MS have been reported in nearly every anatomical site, but is most frequently encountered in skin and soft tissues, lymph nodes, and the gastrointestinal tract [3].

Morphologically, the most common forms of MS are proliferations of immature cells, including myeloblasts, monoblasts, and promonocytes, or less commonly promyelocytes, that efface the normal tissue architecture and are identifiable because of their morphological and immunohistochemical (IHQ) characteristics [4]. The IHQ characterization of the tissue sample is essential in order to establish the definite diagnosis, especially since the tumor may frequently appear without evidence of accompanying Acute Myeloid Leukemia (AML). Usually blast cells express cluster of differentiation 45 (CD45), CD43, and common myeloid markers such as myeloperoxidase (MPO), CD13 or CD33, as well as other markers related with granulocytic or monocytic maturation, such as CD11b or CD14 [5,6]. The differentiation to the erythroid or megakaryocytic line has been exceptionally observed almost always in mixed MS evolved from myeloproliferative syndrome progressions in adult patients [7].

We present the case of a rapidly growing orbital mass in a pediatric female patient with no evidence of morphologic bone marrow involvement, who was finally diagnosed with pure erythroid sarcoma based on histopathology and flow cytometry (FC) criteria. Although the diagnosis of this pathology is usually difficult given the extreme rarity and peculiarity of its presentation, the information obtained by the eight-color standardized FC was key establishing a rapid diagnosis and allowed the prompt start of specific treatment.

A 17-month-old female was admitted to our institution in November 2016. She presented a previous history of nonspecific fever, irritability, and swollen red right eye since September 2016, whereby she was initially diagnosed with cellulitis. Empirical treatment with antibiotics and corticosteroids was started, without improvement.

**Figure 1:**
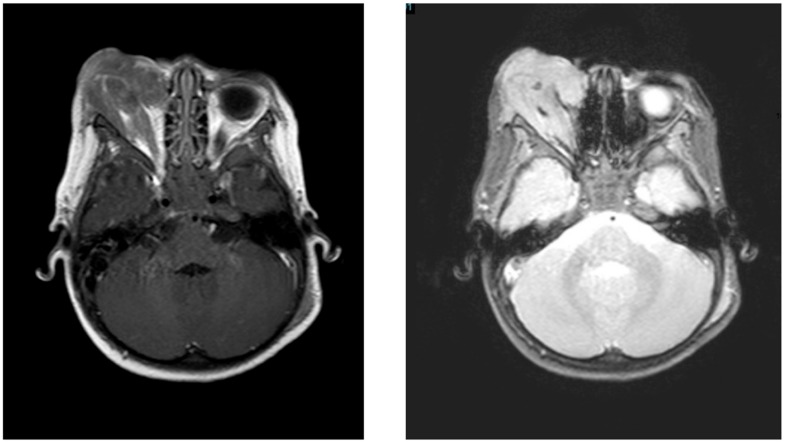
Brain magnetic resonance imaging that shows a prominent mass involving the right orbit. T1 and T2 sequence, respectively.

Physical examination showed the presence of an evident orbital mass with significant ocular proptosis and swollen right face without any other significant findings. Cranial Magnetic Resonance showed an invasive and destructive soft tissue mass (measured 54 × 40 × 15 mm) involving the right orbit, implicating upper jawbone and extending forward to the intraconal space. This mass showed no uniform texture, being mostly iso-intense during T1 and of mildly hyper intense signal during T2 with moderate enhancement after contrast administration (Figure 1). Initial complete blood count showed a hemoglobin level of 10.6 g/dL; a platelet count of 154,000/µL; and a white blood cell (WBC) count of 18,600/µL (10,200/µL lymphocytes). No blasts were seen in the peripheral blood smear. Biochemical tests showed an increase of Lactate Dehydrogenase (LDH) 2414 U/L—normal range <250 U/L. BM aspirate and biopsy did not reveal hematological neoplasia involvement. The cytogenetic study showed a normal female karyotype (46,XX), but the methyltransferase 3 A mutation (variant 512G > A) was detected by a BM molecular study.

**Figure 2:**
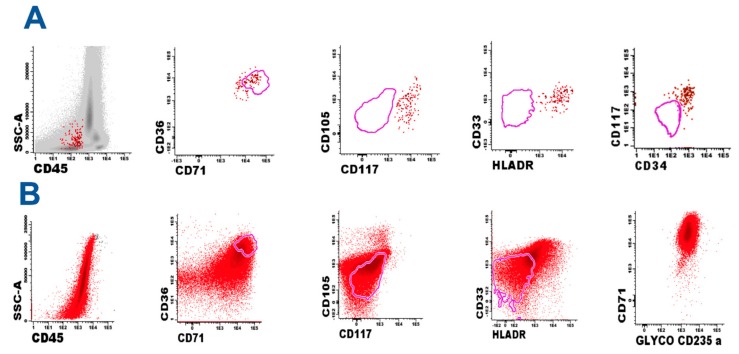
(**A**) Identification of minor population of erythroid leukemic cells (red dots), cluster of differentiation 45^−^ (CD45^−^), CD34^+/−^, CD36^+^, CD71^+^ with abnormal expression of CD105, CD117, Histocompatibility Leukocyte Antigen-D Related (HLADR), and CD33. The correct characterization of these mild changes requires comparison with reference images obtained from normal counterpart (pink line); (**B**) Identification of blast cells in tissue biopsy. Standardized eight-color flow cytometry (FC) demonstrated a homogeneous population similar (not identical) to the previously identified population in a bone marrow sample. Comparison with reference images demonstrated alterations in the expression of CD36, CD71, CD33, and HLADR. The intense homogeneous positivity to glycophorin A demonstrates the pure erythroid nature of the process.

Standardized eight-color FC was performed following EuroFlow protocols [8,9] and 1 million BM cells were analyzed. We were able to characterize a small atypical population consisting of 0.3% of immature erythrocyte precursors (CD36^+^, Histocompatibility Leukocyte Antigen-D Related (HLADR^+^), CD71^+^, CD45^−^, CD105a^+^, CD117 weak with abnormal size and complexity, and aberrant CD33 weak co-expression) (Figure 2A). These cells were highly suspected of corresponding to atypical neoplastic cells. Histological examination showed large undifferentiated monomorphic cellularity with high nucleus-cytoplasm ratios, marked basophilia and frequent mitosis. The IHQ profile included CD43, MPO, CD33, and CD11b as well as pan-B (CD79, CD20), pan-T (CD3, CD5, and CD7), and different epithelial, neural, and muscular markers. The cells showed intense expression of the cellular proliferation-associated antigen Ki-67 (>90% of total cells), weak glycophorin A, moderate expression of CD43, and were negative for the rest of the markers.

**Figure 3:**
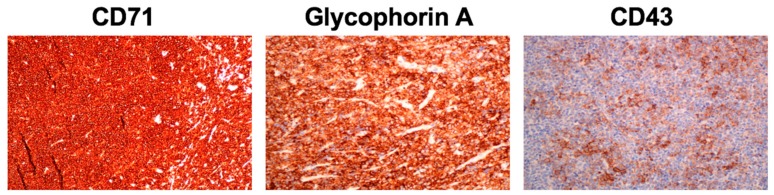
Periorbital biopsy. Diffuse infiltrate of medium-sized cells with intense positivity to CD71 and glycophorin A as well as partial expression of CD43.

Suspecting undifferentiated neoplasia of hematological origin, a mass biopsy was performed and orbital tissue was processed using a gentleMACS dissociator (Miltenyi Biotec, Bergisch Gladbach, Germany) to obtain a single-cell suspension following the manufacturer’s instructions. Standardized eight-color FC demonstrated a homogeneous population of erythroid lineage, with the expression of CD36^+^ weak, CD45^+^, CD105a^+^, CD71^+^, CD117^+^ weak, CD34^+^ weak, and CD33^+^ weak in 100% of the cells. To confirm the erythroid nature of the process, glycophorin A expression was studied (not included in the Euroflow protocol) and intense expression of this marker was observed (Figure 2B). Based on these findings, the IHQ study was completed with the specific markers glycophorin A (Dako M0819, Clone JC 159) and CD71 (Dako f0829, Clone BER-T9), which were specifically indicated for the study of this case, confirming the intense positivity of the proliferating cell population and establishing the erythroid nature of the tumor (Figure 3).

Induction treatment was started according to Spanish Association of Pediatric Hematology and Oncology (SEHOP) AML protocol 2007. After induction, morphological absence of disease was evidenced, with a remarkable decrease in erythroid mass. However, minimal residual disease was detected by high sensitivity standardized eight-color FC, showing the presence of 0.13% erythroid blasts with a similar phenotype to diagnosis (CD36^++^, CD45^+^ weak, CD105a^+^, CD71^+^, CD117^+^, CD34^+^ weak, CD33^+^ weak). After consolidation treatment, resolution of the soft tissue mass component was achieved.

Unfortunately, in March 2017, just before admission to perform allogenic transplantation of hematopoietic stem cell, the ocular lesion progressed with anemia, thrombopenia, and an increase of LDH. The BM aspirate showed a massive infiltration of 90% erythroid blasts (CD36^++^, CD45^+^ weak, CD105a^+^, CD71^+^, CD117^+^ weak, CD34^+^ weak, CD33^−^). A cytogenetic study was performed and showed the presence of a long arm isochromosome of chromosome 7 as well as a balanced translocation between the long arm of chromosome 11 and the long arm of chromosome 20. The obtained karyotype was 46, XX, i (7) (q10), t (11;20) (q13;q11.2).

A second line chemotherapy was initiated without response, and the patient died of progression within one month of relapse.

Pure erythroblastic sarcoma (PES) is a very rare subtype of MS defined as an extramedullar collection of exclusively immature erythroid cells, rarely documented [10,11,12]. Our case represents the fourth patient described in the literature, all of them of pediatric age. Probably due to its exceptionality, PES is not specifically described by the World Health Organization (WHO) Classification of Tumors of Hematopoietic and Lymphoid Tissues book [13], and diagnostic criteria are still not clearly defined.

The few cases have an aggressive evolution as well as certain tropism by the ocular cavity, as happened in our patient. However, this localization is not exclusive. The first documented case was a 3-month-old female with acute erythroid leukemia presenting bilateral ovarian involvement [10]. The second was a 21-month-old female who presented a mass in the left temporal and orbital region, involving the left maxilla and intracranial component [11]. The third was a 3-month-old male presenting left eye proptosis, orbital mass, osteolytic process of the skull, and bone marrow involvement [12].

The major differential diagnosis of myeloid sarcoma is a malignant lymphoma and other solid neoplasms, particularly those that present a diffuse hypercellular histological pattern and poorly differentiated morphology. Given the heterogeneity and rarity of all of these proliferations, it is difficult to establish diagnostic algorithms applicable in the differential diagnosis of these solid neoplasms and many cases are frequently reclassified after an imprecise initial diagnosis [14].

The extraordinary rarity of this pathology and the difficulty of identifying its nature through conventional histopathological and IHQ studies may imply a significant delay in the diagnosis and complicates the patient’s expectations of recovery. In this sense, the BM study using FC and molecular biology are promising diagnostic tools.

FC is a fast and reliable method that should be considered in the diagnosis and quickly provides reproducible results in 2 h or less. Nevertheless, advances in the knowledge of the immunophenotype of erythroid acute neoplasms have been lower than in other leukemias due to the relative scarcity of monoclonal antibodies to identify aberrant patterns as well as the non-application of routinely standardized procedures.

EuroFlow Consortium provides commonly accepted diagnostic algorithms, fully standardized laboratory procedures, and antibody panels in order to achieve maximally comparable results [8,9]. The stability of the experiments also allows a great interlaboratory reproducibility as well as the creation of reference images of great help to identify weak immunophenotypic alterations that can go unnoticed in ordinary FC studies.

Using this methodology, it is very important to carefully investigate the presence of atypical erythroid cellularity, even in apparently normal bone marrow specimens, since varying amounts of atypical erythroid cells can be recognized. On the other hand, the identification of the erythroid lineage in tumor biopsy using eight-color standardized FC has not been previously documented, and is also indispensable for establishing correspondence with BM atypical proliferation and confirming the erythroid nature of a tumor.

In conclusion, the standardized eight-color FC quickly provides reproducible results, and is emerging as a tool to quickly and reliably diagnose this type of malignancy.
